# Three-hour post-ERCP amylase level: a useful indicator for early prediction of post-ERCP pancreatitis

**DOI:** 10.1186/s12876-020-01254-7

**Published:** 2020-04-20

**Authors:** Ze-Hui Lv, Da-Qing Kou, Shi-Bin Guo

**Affiliations:** 1 Department of Gastroenterological Endoscopy, the First Affiliated Hospital of Dalian Medical University, 222 Zhongshan road, Xigang district, Dalian, 116011 Liaoning Province China; 2 Department of Clinical Laboratory, the First Affiliated Hospital of Dalian Medical University, Dalian, 116011 Liaoning Province China

**Keywords:** Endoscopic retrograde Cholangiopancreatography, Serum amylase, Pancreatitis

## Abstract

**Background:**

To evaluate the value of the 3-h post-ERCP serum amylase level for early prediction of post-ERCP pancreatitis (PEP).

**Method:**

A study of 206 patients performed ERCP (Encoscopic Retrograde Cholangio-Pancreatography) at a single centre was done from Jan. 2011 to Nov. 2016. The serum amylase or lipase level was measured at 3 h after ERCP. The patients with PEP were recorded. ROC curves were used to statistically analyze the data: The enrolled patients were divided into two groups according to gender, then we analyzed the data respectively. We comprehensively evaluated the predictive value of PEP by 3-h post-ERCP serum amylase level based on the results above.

**Results:**

Two hundred six patients (92 males, 114 females) were enrolled. PEP occurred in 21 patients (10.19%) among them. The median time to discharge was 7 days (min = 1d, max = 13d) after the procedure. In the 206 patients, the 3-h post-ERCP pancreatic amylase level was used as the test variable, and the PEP occurrence as the state variable to plot the ROC curve. The area under the curve (AUC) was 0.816, and was statistically significant (*P* < 0.001). The standard error (SE) was 0.0507, the 95% confidence interval (CI) was 0.756–0.866, and the optimal cut-off value was 351 U/L (sensitivity 76.19%, specificity 83.24%, positive likelihood ratio 4.55, negative likelihood ratio 0.29, Youden index 59.43%). Of the 206 patients, there were 83 patients with both 3-h post-ERCP amylase level and lipase level detected, and the ROC curves were plotted for both serum amylase and lipase respectively. The ROC curve matched-pair testing was carried out: The areas under the ROC curves were statistically significant. (*P* < 0.001) The area under the ROC curve for the 3-h post-ERCP lipase was 0.778, the 95% confidence interval was 0.673–0.862, and optimal cut-off value was 1834 U/L. The area under the ROC curve for the 3-h post-ERCP serum amylase was 0.780, and the 95% confidence interval was 0.676–0.864. The optimal cut-off is 380 U/L, and there was no statistically significant difference between the two for diagnostic accuracy. According to gender, 206 patients were divided into 2 groups, and the ROC curves were drawn respectively. Based on statistical analysis, there was no statistically significant difference in the diagnostic accuracy of the two groups. In the male group, 436 U/L serum amylase provided the greatest diagnostic accuracy with sensitivity (SE) of 70.5%, specificity (SP) of 89.2%, positive predictive value (PPV) 87.5%, and negative predictive value (NPV) 78.1%. Whereas, in the female group, 357 U/L serum amylase provided the greatest diagnostic accuracy with sensitivity of 76.9%, specificity of 81.2%, positive predictive value of 80.4%, negative predictive value of 77.9%.

**Conclusions:**

1. The 3-h post-ERCP serum amylase level is a useful measurement for predicting post-ERCP pancreatitis. 2. There was no significant difference between serum amylase and lipase 3-h post-ERCP for predicting PEP. 3. There was no statistically significant difference between male and female using the 3-h post-ERCP serum amylase level to predict PEP. For female, the optimal cut-off value was 357 U/L, whereas male 436 U/L .

## Background

Since first reported by McCunne in 1968 [1], ERCP has been widely used in diagnosis and treatment of pancreatic and biliary diseases, especially in the treatment of common bile duct stones. Compared to traditional surgery, it is safer, more effective, with less damage and shorter hospitalization, and has benefits when repeated performance for stone extraction is required [2]. However, its postoperative complications are sometimes difficult to avoid [3]. Post-ERCP pancreatitis (PEP) is one of the most common complications after ERCP procedure. It is reported that the incidence of PEP is 1.6–15.7% [4]. Although 90% are mild or moderate [5], 1% may develop acute necrotizing pancreatitis [6]. Since discharge and readmission after pancreatitis results in worse outcome [7, 8], early diagnosis and timely treatment is very important [9, 10]. So far, the determination of serum amylase is still the most commonly used indicator for the diagnosis of PEP, but there is still no clear standard for the early prediction of PEP at different time points and different levels of amylase [11, 12]. In this study, the ROC curve of the diagnostic test was used to evaluate the value of early prediction of PEP by 3-h post-ERCP serum amylase level.

## Methods

### Patients

From January 2011 to November 2016, a total of 546 patients were diagnosed or treated with ERCP at the First Affiliated Hospital of Dalian Medical University. The study was conducted in compliance with the Helsinki Declaration and in accordance with local legislation, was approved by the Ethics Committee of First Affiliated Hospital, Dalian Medical University. (Ethics References No: YJ-KY-FB-2019-01). Written informed consent was obtained from all of the patients or their relatives before the procedure.

The inclusion and exclusion criteria were as follows:

Inclusion criteria: (1) preoperative serum amylase and lipase levels were normal; (2) age ≥ 18 years; (3) serum amylase level was measured 3 h after ERCP; (4) patients have not used trypsin inhibitor before the diagnosis of PEP.

Exclusion criteria: (1) preoperative diagnosis of acute and chronic pancreatitis (2) abnormal renal function (serum creatinine >92umol / L) (3) pregnant women

According to the inclusion and exclusion criteria above, a total of 206 patients were enrolled, including 92 males and 114 females, among them, 84 cases were simultaneously detected for serum lipase.

The diagnosis of pancreatitis after ERCP is based on the consensus reached by Cotton et al. [4, 13], and the international consensus on the classification of acute pancreatitis in Atlanta in 2012 [14].

1. Acute pancreatic abdominal pain within 24 h after ERCP;
2. Serum amylase more than 3 times the upper limit of normal within 72 h after ERCP (normal value is 30-110 U/L) or lipase greater than 3 times the upper limit of normal within 96 h after ERCP (normal reference range 23-300 U/L);
3. Contrast-enhanced CT, MRI, abdominal ultrasound showing acute pancreatitis changes (pancreatic enlargement, exudation, necrosis and other AP signs); having two of the three criteria will lead to a diagnosis of PEP.
4. The necessity for new or continued hospitalization for at least 2 nights.

### Equipment description

Duodenoscope (JF-260, Olympus Optical Corporation, Tokyo, Japan), guide wire (Hydra Jagwire 0.035 in., Boston Scientific Microvasive. Cork, Ireland), triple lumen sphincterotome (Papillotome, ENDO-FLEX GmbH, Germany), CRE balloon catheter (Boston Scientific Microvasive, Cork, Ireland), retrieval balloon catheter (Extractor Three Lumen Retrieval Balloon, Boston Scientific Microvasive. Cork, Ireland), Dormia basket (Web™ extraction basket, Wilson-Cook Medical Inc. Winston-Salem, North Carolina, United States), Percuflex Biliary Stent (Boston Scientific Corporation,One Boston Scientific Place, Natick, MA 01760–1537, USA), nasal biliary drainage tube (nasobil. Sonde, ENDO-FLEX GmbH, Germany), WallFlex Biliary RX Fully Covered Stent System (Boston Scientific Corporation,One Boston Scientific Place, Natick, MA 01760–1537, USA),Mechanical lithotripsy (BML-4Q; Olympus Optical, Tokyo, Japan).

Some ERCP procedures were accomplished under ECG monitoring. Tetracaine was given for local anesthesia of the pharynx. The patients were sedated and relieved pain by intramuscular administration of diazepam and meperidine. Twenty mg of butyl scopolamine bromide was injected intramuscularly prior to the procedure to inhibit duodenal peristalsis.

A sphincterotome with a guide wire was used for selective cannulation. Difficulties in selective cannulation including stenosis and sclerosis of the papillary, incarceration of common bile duct (CBD) stone at papilla, and periampullary diverticulum, especially the papilla is located at bottom of the diverticulum.

For some cases difficulty in bile cannulation, precut through pancreatic duct was applicated. For cases with incarceration of CBD stone at papilla, needle knife was used for precut sphincterotomy. For papilla located at the bottom of diverticulum, it was exposed by eversion diverticulum through biopsy forceps, or fixation by metal clip, or submucosal injection of saline.

For patients with CBD stones, small endoscopic sphinecterotomy (EST) alone or EST combined with endoscopic papillary balloon dilation (EPBD) was performed. For biliary tract benign stricture or biliary fistula, ERBD was given. For biliary or pancreatic malignant tumor, a self-expanding metal stent was placed.

Mechanical lithotripsy was used for large common bile duct stones. However, if patient was in poor condition and cannot tolerate long procedure time, just ERBD was placed for drainage and to relieve symptoms. And a next ERCP was performed for removal of cholelithiasis 3 months later.

Serum amylase, lipase, biochemical liver function and renal function were detected by Johnson & Johnson’s VITROS FS5.1 automatic biochemical analyzer.

### Statistical methods

According to the inclusion and exclusion criteria above, a total of 206 patients were enrolled, and each patient’s progress note was reviewed by the medical record system. Sudden pancreatitis-like mid-abdominal pain within 24 h after ERCP was marked positive, otherwise negative; combined with CT, serum amylase, lipase levels, comprehensive estimation was made according to the PEP diagnostic criteria .

#### Statistical methods

1. Descriptive analysis of the enrolled patients, a preliminary understanding of the basic situations of the study objects. 2. The ROC curve was plotted according to the 3-h post-ERCP serum amylase level of the enrolled patients and PEP diagnosis. According to the area under the ROC curve and the 95% confidence interval of the area, the accuracy of the tests was evaluate based on statistical tests. The Youden index was calculated according to the sensitivity and specificity of each level and the optimal cut-off value was obtained. The sensitivity, specificity, positive and negative predictive value, positive and negative likelihood ratio, Youden index of different cut-off sites were calculated, comprehensively evaluated the accuracy and predictive value of the predictive diagnostic method. 3. According to gender, they were divided into two groups, and the ROC curves were drawn and analyzed separately aimed to find best cut-off in different gender. As a same lab test whose diagnosis accuracy (be showed as AUC in ROC) should have no difference between genders or other classifications of subjects. So we use Z test to test the reliability of data, then obtain the optimal diagnostic cut-off value according to these two curves. 4. Matched-pair analysis of ROC curves in patients who were simultaneously tested for both serum amylase levels and lipase levels 3 h after ERCP. According to the two test Methods, the ROC curves were drawn and statistically tested, and the difference test (Z test) was performed to compare and analyze the diagnostic accuracy and diagnostic value in the two groups.

#### Statistical software

Medcalc was applied to perform matched-pair analysis. The ROC curve of serum amylase of different gender patients were plotted using SPSS software, and then the difference test was performed according to the standard error and the area under the curve. The Z value was manually calculated, and the *P* value was looked up in the table .

Related formula:

Sensitivity: Se; Specificity: Sp.

1 $$ \mathrm{Positive}\ \mathrm{likelihood}\ \mathrm{ratio}:\kern0.5em \mathrm{LR}\left(+\right)=\frac{\mathrm{Se}}{1\hbox{-} \mathrm{Sp}} $$

2 $$ \mathrm{Negative}\ \mathrm{likelihood}\ \mathrm{ratio}:\kern0.5em \mathrm{LR}\left(-\right)=\frac{1\hbox{-} \mathrm{Se}}{\mathrm{Sp}} $$

3 $$ \mathrm{Positive}\ \mathrm{p}\mathrm{redictive}\ \mathrm{value}:\kern0.5em \mathrm{PPV}=\frac{\mathrm{p}\ast \mathrm{Se}}{\mathrm{p}\ast \mathrm{Se}+\left(1\hbox{-} \mathrm{p}\right)\ast \left(1\hbox{-} \mathrm{Sp}\right)}\times 100\% $$

4 $$ \mathrm{Negative}\ \mathrm{predictive}\ \mathrm{value}:\mathrm{NPV}=\frac{\left(1-\mathrm{p}\right)\ast \mathrm{Sp}}{\left(1-\mathrm{p}\right)\ast \mathrm{Sp}+\mathrm{p}\ast \left(1-\mathrm{Se}\right)}\times 100\% $$

5 $$ \mathrm{Youden}\ \mathrm{Index}:\kern0.5em \mathrm{YI}=\mathrm{Se}+\mathrm{Sp}\hbox{-} 1 $$

## Results

### Basic situations

A total of 562 patients who underwent diagnostic or therapeutic ERCP in our hospital between January 2011 to November 2016. The total number of patients who met the criteria for enrollment was 206, 92 males and 114 females. Among the 206 patients, endoscopic sphinecterotomy (including EST alone and EST combined with EPBD) was performed in 136 patients; endoscopic nasobiliary drainage (ENBD) was performed in 140 patients; endoscopic retrograde biliary drainage (ERBD) was performed in 36 patients (Table 1). The median duration from the procedure to discharge was 7 days (min = 1d, max = 13d). A total of 21 patients (10.19%) were diagnosed with PEP, 2 patients (0.97%) had upper gastrointestinal bleeding, and 3 patients (1.45%) had post-ERCP infection. No case of perforation.

**Table 1:** Basic information of patients who had ERCP

variable	number
Age	
< 50y	45
50~60y	47
61~70y	57
71~80y	44
> 80y	13
Gender	
Male	92
Female	114
Context	
Suspected or known stone	165
Biliary tract benign stricture	6
Biliary fistula	3
Biliary or pancreatic tumor	31
Intraductal papillary mucinous neoplasm	1
History of pancreatitis (including PEP)	4
Jaundice	112
Priority	
Urgent	17
Elective	189
Therapy	
EST alone	58
Small EST + EPBD	78
EPBD alone	9
ENBD	140
ERBD	32
Mechanical lithotripsy	12
EMBE	28
ERPD	28

The average amylase level was 224.44 U/L in the non-PEP group, with a median of 115.00 U/L. The average amylase level in the PEP group was 688.90 U/L, with a median of 456 U/L. The serum amylase levels in the PEP group and the non-PEP group after ERCP were positively skew distribution, and the results showed that the serum amylase levels 3-h after ERCP were significantly higher than those of the non-PEP group (non-PEP group stem width = 100; PEP group stem width = 1000).

### Analyses of ROC curve of amylase level 3-h after ERCP and PEP diagnosis in 206 patients

**Fig. 1:**
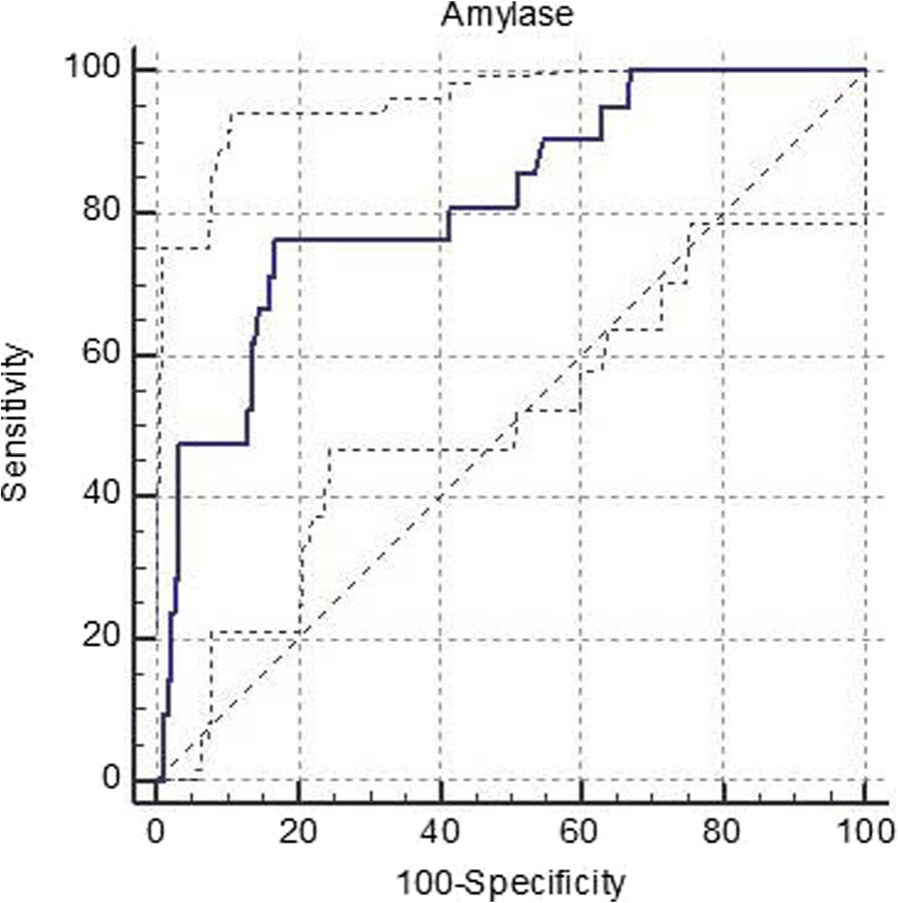
ROC curve of amylase level 3-h after ERCP and PEP diagnosis in 206 patients

The non-parametric method was used in this paper, the serum amylase level 3-h post-ERCP as the test variable, the PEP occurrence as the state variable and the ROC curve was drawn. The result is shown in Fig. 1 (the solid line is the drawn ROC curve, and the dotted line is 95% confidence interval), In Tables 2, the area under the ROC curve was 0.816, the diagnostic value is good, the standard error was 0.0507, the 95% confidence interval was 0.756–0.866, and the significance test Z value was 6.235, *P* < 0.0001. The area under the ROC curve was statistically significant, and the optimal cut-off value was 351 U/L. The sensitivity, specificity, positive and negative likelihood ratio, positive and negative predictive value, and Youden index of different cut-off values are shown in Table 3:

**Table 2:** ROC area under curve (AUC)

ROC area under curve (AUC)	0.816
Standard error	0.0507
95% confidence interval (CI)	0.756–0.866
Z score	6.235
*P* value (area = 0.5)	< 0.0001

**Table 3:** Youden Index

Youden index J	0.5943
Related standards	> 351
sensitivity	76.19
specificity	83.24

As seen from the Table 4, when 351 U/L was taken as the optimal cut-off value, a better positive predictive value (81.90) could be obtained, the Youden index was the highest (59.43%), and the diagnosis was the most accurate at this time. Meanwhile, the positive and the negative likelihood ratio were both high (4.55, 0.29), indicating that the possibility of diagnosis or elimination of the disease was high. When taking 105 U/L (close to the standard upper limit of normal), the negative likelihood ratio reached 0.21, which indicates that this is an ideal diagnostic criterion with a favorable negative predictive value and has certain clinical reference significance.

**Table 4:** Different thresholds in diagnosis for 206 patients

cut-off value(U/L)	SE(%)	SP(%)	+LR	-LR	PPV(%)	NPV(%)	YI(%)
> 105	90.48	45.41	1.66	0.21	62.37	82.67	35.98
> 167	76.19	66.49	2.27	0.36	69.45	73.63	42.68
> 229	76.19	71.89	2.71	0.33	73.05	75.12	48.08
> 326	76.19	81.08	4.03	0.29	80.11	77.30	57.27
> 447	57.14	86.49	4.23	0.50	80.88	66.87	43.63
Optimal cut-off(351 U/L)	76.19	83.24	4.55	0.29	81.90	77.76	59.43

### ROC curve matched-pair testing for the 3-h post-ERCP amylase level between male and female, and further analysis according to gender

**Fig. 2:**
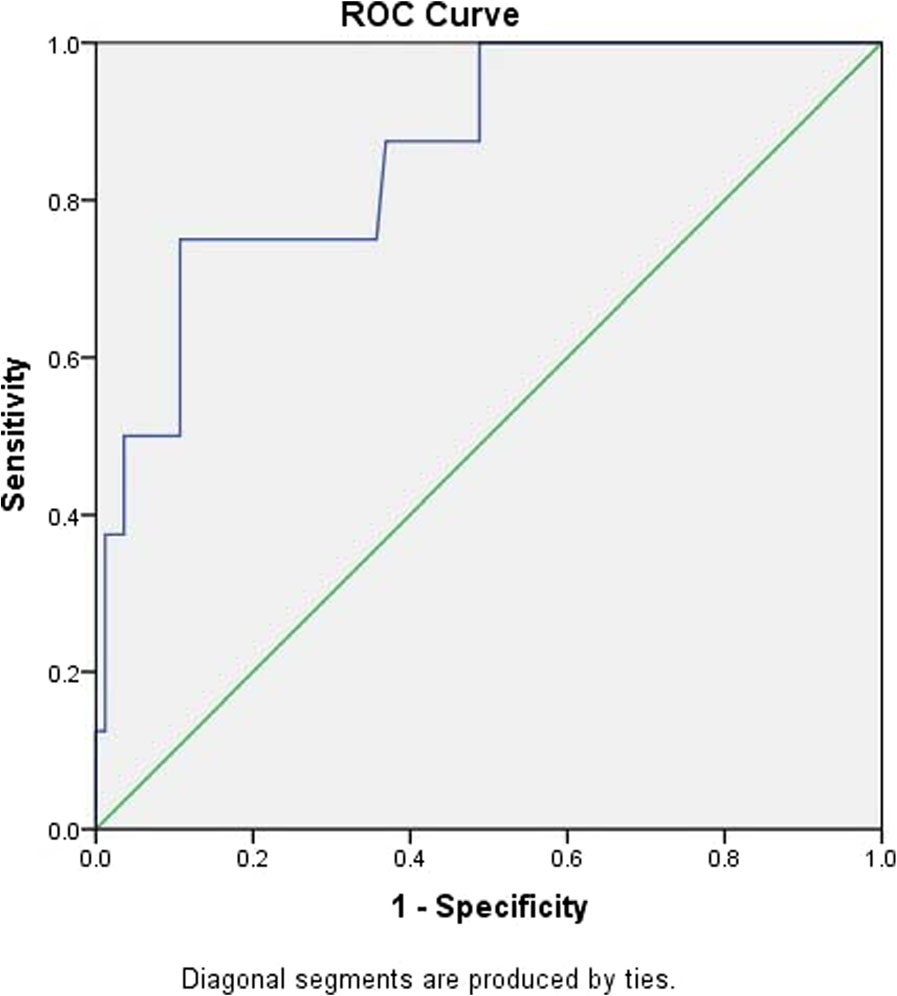
ROC curve of amylase level 3-h after ERCP and PEP diagnosis in male group

**Fig. 3:**
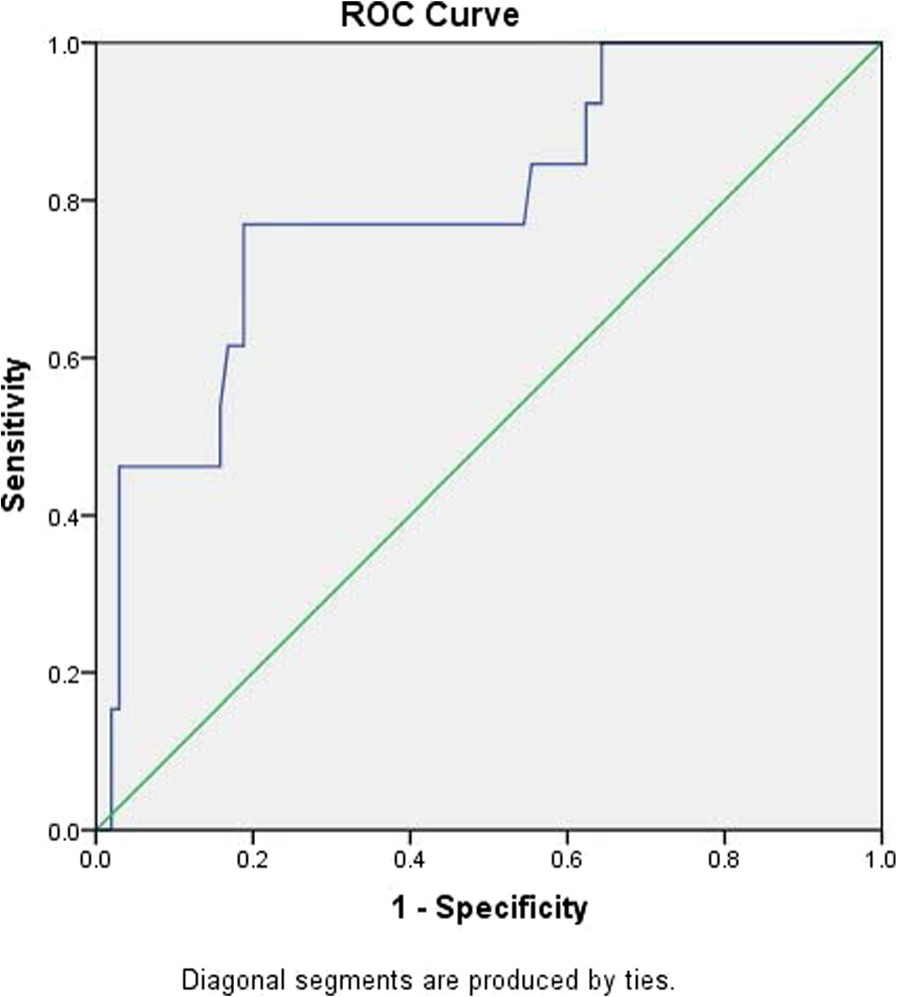
ROC curve of amylase level 3-h after ERCP and PEP diagnosis in female group

In order to investigate whether different genders groups need different diagnostic criteria, ROC curve was applied to evaluate this issue in our study. Two hundred six patients were divided into two groups according to gender, and the ROC curves were plotted respectively(Fig. 2, male; Fig. 3, female). The results are shown as follows:

The ROC curves drawn for the two groups both had statistical significance (*P* = 0.001 for both groups), the area under the curve for the male group was 0.859, the standard error was 0.065, and the area under the curve for the female group was 0.794, and the standard error was 0.068 (Tables 5,6). It indicated that the accuracies of the two tests were good; the two groups were independent samples, suitable for comparison of independent ROC curves to test the statistical significance. The Z value of the hypothesis test was 0.69098, less than 1.96, *P* > 0.05, which indicated there was no statistical difference between the two groups. We know that the ROC curve is not affected by the prevalence rate. The results indicate that although the prevalence rates were different in the two groups, there was no statistical difference in the accuracy of predicting PEP by amylase level 3-h post-ERCP between the two groups, which also verifies the validity of our study.

**Table 5:** Predictive value of amylase detection in male group

ROC area under curve (AUC)	Standard error	P value	95% confidence interval (CI)
0.859	0.065	0.01	0.733–0.986

**Table 6:** Predictive value of amylase detection in female group

ROC area under curve (AUC)	Standard error	*P* value	95% confidence interval (CI)
0.794	0.068	0.01	0.662–0.927

The corresponding diagnostic values according to the optimal cut-off values are shown in the following Table 7: In the male group, 436 U/L serum amylase provided the greatest diagnostic accuracy with sensitivity of 70.5%, specificity of 89.2%, positive predictive value of 87.5%, and negative predictive value of 78.1%. Whereas, in the female group, 357 U/L serum amylase provided the greatest diagnostic accuracy with sensitivity of 76.9%, specificity of 81.2%, positive predictive value of 80.4%, negative predictive value of 77.9%. The NPV in the male group was higher than that of the female group, which indicated that the proportion of PEP in the male group was also higher than that of the female group. This was consistent with the objective situation that women have a higher incidence of PEP than men.

**Table 7:** Comparison of male and female groups

	Male	Female
AUC	0.859	0.794
Selected cut-off	436 U/L	357 U/L
SP	0.892	0.812
SE	0.750	0.769
PPV	0.875	0.804
NPV	0.781	0.779
YI	0.643	0.581

### Matched-pair analysis of the 3-h post-ERCP serum amylase and lipase levels

**Fig. 4:**
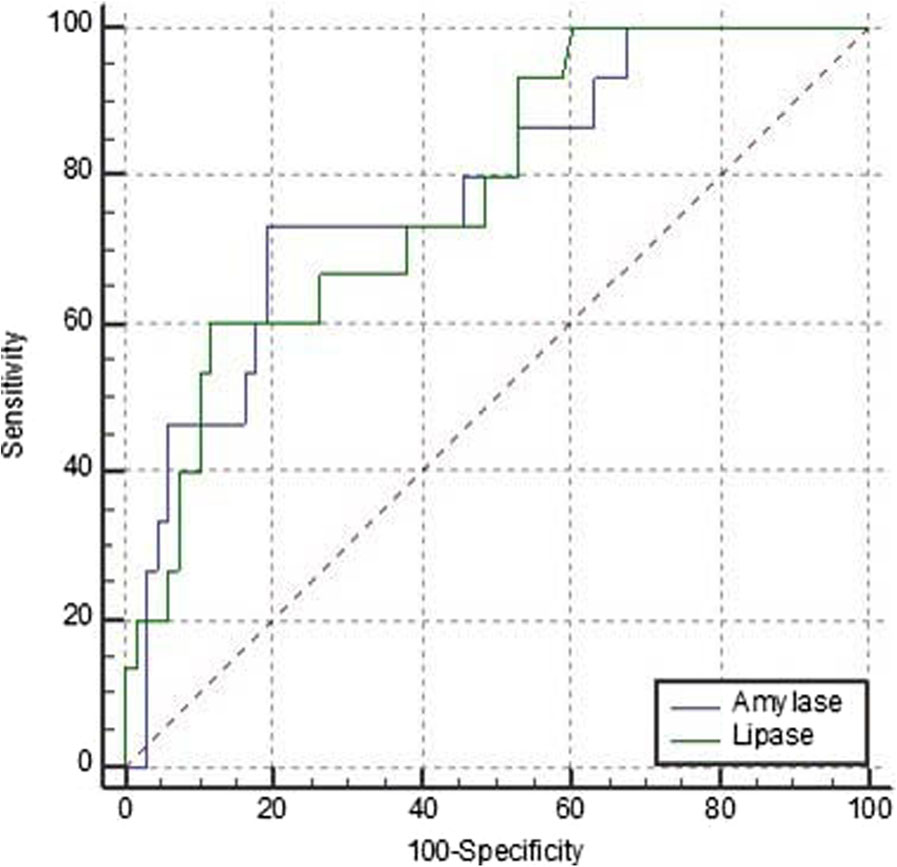
ROC curve of the 3-h post-ERCP serum amylase, lipase levels and PEP diagnosis

The ROC curve matched-pair testing was used to compare the serum amylase and serum lipase 3 h after ERCP. A total of 83 patients with both the serum amylase and lipase levels 3-h post-ERCP were selected. Among them, 15 patients were diagnosed as PEP. The ROC curves were drawn (Fig. 4) and the results were compared as follows: The ROC curves of the two groups were statistically significant (*P* < 0.001). The area under the ROC curve of lipase levels 3-h post-ERCP was 0.778, with a 95% confidence interval of 0.673–0.862; The area under the ROC curve of amylase 3-h post-ERCP was 0.780, and the 95% confidence interval was 0.676–0.864, indicating that the accuracy of predicting PEP was high (Table 8). Matched-pair testing was performed between the two groups, and the results showed that there was no statistically significant difference in the accuracy of predicting PEP (Z score 0.0598, *P* = 0.9523, *P* > 0.05) (Table 9), and the negative predictive value of lipase 3-h post-ERCP was not high (0.688) (Table 10).

**Table 8:** Statistical test of lipase and amylase

Variables	AUC	SE	95% CI
Amylase	0.780	0.0667	0.676–0.864
Diagnosis	1.000	0.000	0.957–1.000
Lipase	0.778	0.0639	0.673–0.862

**Table 9:** ROC curve matched-pair testing of amylase and lipase

Amylase vs Lipase	
Difference between areas	0.00245
Standard error	0.0410
95% CI	−0.0779 - 0.0828
Z score	0.0598
Statistical significance	P = 0.9523

**Table 10:** Diagnostic indicators of lipase and amylase

	The 3-h post-PEP serum amylase	The 3-h post-PEP serum lipase
AUC	0.778	0.780
The optimal cut-off value	1834 U/L	380 U/L
Sp(%)	88.2	80.9
Se(%)	60.0	73.3
PPV(%)	83.6	79.3
NPV(%)	68.8	75.2
YI(%)	48.2	54.2

## Discussion

ERCP has become an important method in clinical diagnosis and treatment for biliary and pancreatic diseases. However, its complications should not be ignored, especially post-ERCP pancreatitis (PEP). As the most common post-ERCP complication, PEP has restricted the development of ERCP. The average incidence of PEP is reported to be 3.5% in the literatures, but in high-risk groups (such as previous PEP, difficulty in cannulation of the bile duct, etc.), the incidence can be as high as 30% [5]. Although most cases of PEP were mild [5], they recovered after conservative treatment in less than 72 h, in some severe cases, the length of hospital stay can be extended, the costs can be increased, multiple organ failure may occur, even death [6]. Since it is difficult to avoid PEP even if the procedure is performed by an endoscopic expert, early detection and timely treatment for the PEP are very important. Serum amylase measurement technology is simple, easy to obtain for low price with high sensitivity and early appearance. Although the level of amylase cannot estimate the severity of pancreatitis, it is still one of the most commonly used indicators for diagnosis of PEP currently. However, there is still no clear criteria for various cut-off serum amylase levels at different time points in prediction and diagnosis of PEP [9, 11, 15–18].

The role of serum amylase levels in predicting the occurrence of PEP after ERCP has been recognized [19]. Takayoshi Nishino [16] et al. have demonstrated a good negative predictive value of the serum amylase levels 4-h post-ERCP for PEP (AUC 0.91, *p* = 0.007) through a retrospective study of 1631 patients. Kapetanos et al. [20] also have demonstrated that the 2-h post-ERCP amylase cut-off value 3 times the upper limit of normal exhibited an NPV and a PPV for PEP of 95 and 32% respectively, which indicates the 2-h post-ERCP serum amylase is valuable for eliminating the diagnosis of PEP. However, it can not be used for early detection of the PEP. Studies have shown that serum amylase levels at 6, 8, and 12 h after ERCP have a good predictive effect on PEP, and the accuracy of prediction is higher over time, but the significance of early prediction has been lost [21]. The studies by Sutton et al. [12] suggest that serum amylase value 4-h after ERCP greater than 5 times the upper limit of normal (ULN), or serum amylase value greater than 2.5 times the ULN and pancreatic duct visualization are good predictors of PEP.

Our study showed that the serum amylase level 3-h after ERCP has a good accuracy in the prediction of PEP. The cut-off at 351 U/L provides the optimal diagnostic accuracy, when setting the cut-off at 105 U / L (close to the standard upper limit of normal), the negative likelihood ratio can reach 0.21, indicating that this is a valuable predictive value for eliminating PEP.

Studies have confirmed that female gender is one of the important risk factors for pancreatitis after ERCP [22], so whether different genders need to use different predictive diagnostic criteria is one of the purposes of this study.By using the ROC curve matched-pair testing, the results showed that the two groups have the best diagnostic accuracy at different cut-off values. For female, the optimal cut-off value was 357 U/L, whereas male 436 U/L. This conclusion may provide help for clinicians. In addition, nearly 25% of patients with type 2 diabetes have elevated levels of lipase and/or amylase without the symptoms of acute pancreatitis [23]. Whether we need to establish different cut-off values for the diagnosis of PEP to obtain a better predictive effect for those patients, is still to be validated by more clinical studies.

Serum lipase is a specific diagnostic indicator of pancreatitis. Its activity is maintained longer than amylase, and there is no other source in the blood [24], so it is considered to be more sensitive than serum amylase (95%;79%). The British guidelines for the diagnosis and treatment of acute pancreatitis in 2005 indicated that the pancreas is the only source of lipase and is superior to amylase in terms of sensitivity, specificity and accuracy [25]. And the American guidelines for the diagnosis and treatment of acute pancreatitis in 2006 also had similar views [26]. The European Guidelines for Digestive Endoscopy in 2014 clearly indicated that serum amylase < 1.5*ULN or serum lipase< 4*ULN monitored 2–4 h after ERCP provide a high NPV for PEP, and the patients can be discharged without considering the risk of PEP (recommended level B) [19], which suggests that serum lipase has a high negative predictive value for PEP. Gumaste et al. [27] pointed out based on an analysis of 170 patients with abdominal pain, the negative predictive value for acute pancreatitis was 98%. However, we emphasized the early (3-h) lipase and amylase levels after ERCP in this study. And the results showed that in terms of comparison the predictive value for PEP between serum lipase and amylase 3-h post-ERCP, lipase was not better than amylase. It is well known that serum lipase starts to rise at 24-48 h and reaches a peak at 72–96 h and last for 7-10d. This seems to explain why the early lipase monitoring value is not significantly better than that of amylase. Based on the actual situations in China, the predictive value of the joint monitoring needs to be further verified, and the economic benefits of the two tests need further cost-benefit analysis.

However, this was a retrospective study in a single center, and small sample was another limitation for the study. More cases are required to confirm the value of the 3-h post-ERCP serum amylase level for early prediction of post-ERCP pancreatitis in the future.

## Conclusions

In summary, we have come to the following conclusions:

The 3-h post-ERCP serum amylase level is a useful measurement for early predicting post-ERCP pancreatitis. With the 3-h post-ERCP serum amylase level 1–1.5 times upper limit of normal, the negative predictive value is high.

There was no significant difference between serum amylase and lipase 3-h post-ERCP for predicting PEP.

There was no statistical difference between male and female using the 3-h post-ERCP serum amylase level for prediction PEP. For female, the optimal cut-off value was 357 U/L, whereas male 436 U/L.

## Data Availability

Data is available from the corresponding author.

## Abbreviations

PEP: Post-ERCP pancreatitis
AUC: Area under the curve
SE: Standard error
CI: Confidence interval
SE: Sensitivity
SP: Specificity
PPV: Positive predictive value
NPV: Negative predictive value
ERCP: Endoscopic retrograde cholangiopancreatography
EST: Endoscopic sphinecterotomy
ENBD: Endoscopic nasobiliary drainage
ERBD: Endoscopic retrograde biliary drainage
EPBD: Endoscopic papillary balloon dilation
ERPD: Endoscopic retrograde pancreatic drainage
EMBE: Endoscopic metal biliary endoprothesis
ULN: Upper limit of normal
